# Concurrent sintilimab with sequential chemoradiotherapy for unresectable, stage III non-small cell lung cancer: a retrospective study

**DOI:** 10.3389/fonc.2023.1129989

**Published:** 2023-04-20

**Authors:** Shi Tang, Xiaofeng Cong, Dan Zheng, Chen Chen, Zengguang Liu, Jie Gao, Huimin Zhang, Youhao Zhang, Ziling Liu

**Affiliations:** Cancer Center, The First Hospital of Jilin University, Changchun, China

**Keywords:** non-small cell lung cancer, chemoradiotherapy, PD1/PDL1 inhibitor, sintilimab, safety, retrospective study

## Abstract

**Background:**

Concurrent programmed death 1 (PD-1) or programmed death ligand 1 (PD-L1) inhibitors with sequential chemoradiotherapy (SCRT) have been reported in only a limited number of studies involving patients with unresectable stage III non-small-cell lung cancer (NSCLC). A retrospective study was conducted to systematically analyze the efficacy and safety of the emerging therapy among Chinese patients.

**Materials and methods:**

We included patients with unresectable, stage III NSCLC who received concurrent sintilimab with chemotherapy or chemotherapy alone for 3-6 cycles, followed by radical radiotherapy at the First Hospital of Jilin University from Dec 15, 2019, to Jul 15, 2022. The primary end point was the objective response rate (ORR). The secondary end points included progression-free survival (PFS), overall survival (OS), 12-month and 18-month PFS rates, the duration of response (DoR), and safety.

**Results:**

The retrospective study involved 77 patients, of which 49 receiving concurrent sintilimab with SCRT were assigned to cohort A, and 28 receiving SCRT alone were assigned to cohort B. The ORR was significantly higher in cohort A (79.6%, 95% CI 65.7–89.8) than in cohort B (35.7%, 95% CI 18.6–55.9) (p<0.001). Median PFS was significantly longer in cohort A than in cohort B (NR [95% CI 21.4–NR] vs. 16.0 months [13.0–22.5]; HR 0.375, 95% CI 0.192–0.735; p=0.003). The PFS rates at 12 and 18 months were 84.8% (95% CI 75.0–95.9) and 71.3% (95% CI 58.7–86.7) in cohort A and 75.0% (95% CI 60.6–92.9) and 38.3% (95% CI 23.7–61.7) in cohort B, respectively. Grade 3 or 4 adverse events (AEs) were reported in 19 patients (38.8%) and seven patients (25.0%) in two cohorts, respectively. Grade 3 or 4 pneumonitis or immune-mediated pneumonitis, radiation pneumonitis, and pneumonia occurred in five (10.2%), four (8.2%), and two (4.1%) cohort A patients, and zero, two (7.1%), and two (7.1%) cohort B patients, respectively. Only cohort A reported AE leading to death in one (2.0%) patient (immune-mediated pneumonitis).

**Conclusion:**

Concurrent sintilimab with SCRT resulted in a significantly better ORR and longer PFS than SCRT alone, with manageable safety profiles in Chinese patients with unresectable stage III NSCLC.

## 1 Introduction

Lung cancer is the leading cause of cancer death worldwide, with an estimated 1.8 million deaths in 2020 (1). NSCLC accounts for approximately 85% of lung cancers. About one-third of patients have stage IIIA-IIIC, a locally advanced disease at diagnosis (2, 3). Approximately 30%-50% of individuals with stage III NSCLC are inoperable at diagnosis (4, 5). Concurrent chemoradiotherapy (CCRT) is the standard of care for patients with unresectable stage III NSCLC without significant breakthroughs for many years (6–14). SCRT has been recommended in international treatment guidelines as an alternative for unresectable stage III NSCLC patients who cannot access or tolerate CCRT (15–17). Recently, immune checkpoint inhibitors have become a revolutionary treatment for patients with unresectable stage III NSCLC.

In the placebo-controlled phase III PACIFIC trial, durvalumab as consolidation therapy in patients with unresectable stage III NSCLC treated with definitive CCRT significantly improved PFS and OS (18, 19). As consolidation therapy in the GEMSTONE-301 study, sugemalimab after CCRT or SCRT showed promising efficacy in Chinese patients with unresectable stage III NSCLC (20). However, some patients suffered from disease progression (PD) during CCRT or SCRT, thereby could not benefit from the PD-1/PD-L1 inhibitors as consolidation therapy. Researchers are beginning to explore the efficacy and safety of concurrent PD-1/PD-L1 inhibitors with chemoradiotherapy therapy, as it would benefit a larger patient population. Most relevant trials focus on CCRT until now (21–28). However, the CCRT application in clinical practice has been limited, mainly due to the toxicity, comorbidities, advanced age, or frailty of patients, tumor volumes and locations, and the lack of relevant facilities (29, 30). SCRT is a common and effective alternative to CCRT in clinical practice because it is associated with potentially lower radiotherapy volumes, lower toxicity, and ease of planning in clinical practice.

Sintilimab, a fully human IgG4 monoclonal antibody, can inhibit the interaction between PD-1 and its ligands, thereby restoring the endogenous anti-tumour T-cell response by selectively binding to PD-1 (31). The combination of sintilimab and chemotherapy revealed excellent efficacy and manageable safety profile for patients with advanced or metastatic squamous and non-squamous NSCLC in the ORIENT-12 and ORIENT-11 trials, respectively (32, 33). Most patients receive SCRT in clinical practice in China, demanding outcome improvement. Few published studies have systematically examined the efficacy and safety of concurrent PD-1/PD-L1 inhibitors with SCRT. We conducted a real-world retrospective study to systematically analyze the efficacy and safety of concurrent sintilimab with SCRT in patients with unresectable, stage III NSCLC.

## 2 Materials and methods

### 2.1 Patients

Eligible patients were at least 18 years old with histologically or cytologically confirmed stage IIIA-IIIC, unresectable NSCLC [based on the eighth edition of TNM classification by the International Association for the Study of Lung Cancer Staging project (34)]; intolerant of CCRT; had received concurrent sintilimab with chemotherapy or chemotherapy alone for 3-6 cycles followed by radiotherapy at the First Hospital of Jilin University from Dec 15, 2019, to Jul 15, 2022; had at least one measurable lesion following the Response Evaluation Criteria in Solid Tumors version 1.1 (RECIST v1.1); and had an Eastern Cooperative Oncology Group (ECOG) performance status score of 0 or 1. Patients were excluded if they had more than one primary tumor; serious concomitant diseases or active infections; a history of primary immunodeficiency or active autoimmune disease; or symptomatic interstitial lung disease. **Supplementary Figure 1** represents the flow chart for patient enrollment.

Patients were stratified by age (<62 or ≥62 years), sex (male or female), smoking status (former/current smoker or never smoked), ECOG performance status score (0 or 1), disease stage (IIIA, IIIB or IIIC), tumor histological type (squamous cell carcinoma or non-squamous cell carcinoma), radiotherapy dose (54-59Gy or 60-66Gy) and induction therapy cycles (3-4 cycles or 5-6 cycles). Patients who received concurrent sintilimab with SCRT were assigned to cohort A, and those who received SCRT alone were assigned to cohort B. The First Hospital of Jilin University Ethics Committee approved the research protocol. All patients or their next of kin provided written informed consent for using their data before receiving treatment.

### 2.2 Treatments

We intravenously (IV) administered 200 mg sintilimab and platinum-based chemotherapy or platinum-based chemotherapy alone for 3-6 cycles as an induction therapy on day 1 of each cycle, once every three weeks (Q3W), followed by radical radiotherapy at 54-66Gy. Sintilimab 200 mg was administered as consolidation therapy every three weeks for two years or until PD. The platinum-based chemotherapy regimens administered were as follows: (1)albumin-bound paclitaxel (260mg/m² IV on day 1 Q3W)+carboplatin (area under the concentration-time curve, 5 mg/mL/min IV on day 1 Q3W)/cisplatin (75 mg/m² IV on day 1 Q3W); (2)pemetrexed (500 mg/m² IV on day 1 Q3W)+carboplatin/cisplatin (same as protocol 1); (3)docetaxel (75mg/m² IV on day 1 Q3W)+carboplatin/cisplatin (same as protocol 1); (4)gemcitabine (1.0 g/m² IV on day 1 and day 8 Q3W)+carboplatin/cisplatin (same as protocol 1).

### 2.3 Assessment and end points

A contrast-enhanced computed tomography scan was conducted at the baseline and performed once every six weeks until consolidation therapy and every 12 weeks afterward. The response was assessed using RECIST 1.1. The AEs were graded based on the National Cancer Institute Common Terminology Criteria for Adverse Events (CTCAE).

The primary end point was the ORR [defined as the proportion of patients whose best response was complete response (CR) or partial response (PR)]. The secondary end points included PFS (defined as the time from the beginning of treatment to disease progression or death in the absence of disease progression); OS (defined as the time from the beginning of treatment to death due to any cause); 12-month and 18-month PFS rates; the DoR (defined as the time from the first documented CR or PR to PD or death); and safety.

### 2.4 Statistical analysis

The chi-square and Fisher’s exact tests were used to assess the differences between baseline characteristics. The Kaplan-Meier method was used to estimate the PFS, OS, and DoR while determining the PFS and OS rates at different time points. The log-rank test was used to compare the difference in treatment. The hazard ratio (HR) and 95% confidence intervals (CIs) were calculated using a stratified Cox regression model. The HR and 95% CI were estimated with an unstratified Cox regression model with the treatment covariate in prespecified subgroups. The Clopper-Pearson method was used to estimate the 95% CI for ORR in each cohort. Differences in ORR between the two cohorts were assessed using Fisher’s exact test. R 4.2.0 software was used for statistical analyses, and values were considered significant if P<0.05.

## 3 Results

### 3.1 Patients

A total of 77 patients were included in this retrospective study, among which 49 patients receiving concurrent sintilimab with SCRT were assigned to cohort A, and 28 patients receiving SCRT alone were assigned to cohort B. The two cohorts had adequately balanced baseline characteristics (**Table 1**). The median ages in cohorts A and B were 62 years (range: 45–75 years) and 59 years (range: 46–71 years), respectively. The demographic characteristics of patients revealed that most patients were male (81.8%) and former or current smokers (79.2%), with an ECOG performance status of 1 (58.4%). Most patients had presented with stage IIIA (45.5%) and a majority had tumors of the squamous histologic type (75.3%). Many patients received induction treatment for 5-6 cycles (51.9%) and a radiotherapy dose of 60-66Gy (77.9%).

**Table 1:** Baseline characteristics of all patients.

Variables	Total population	Cohort A	Cohort B	P value†
	N=77 (%)	N=49 (%)	N=28 (%)	
Sex				
Male	63 (81.8)	41 (83.7)	22 (78.6)	0.577
Female	14 (18.2)	8 (16.3)	6 (21.4)	
Age (years)				
Median	61	62	59	
<62	40 (67.5)	23 (65.3)	17 (71.4)	0.244
≥62	37 (32.5)	26 (34.7)	11 (28.6)	
Smoking status				
Never smoked	16 (20.8)	12 (24.5)	4 (14.3)	0.441
Former/current smoker	61 (79.2)	37 (75.5)	24 (85.7)	
ECOG PS				
0	32 (41.6)	20 (40.8)	12 (42.9)	0.861
1	45 (58.4)	29 (59.2)	16 (57.1)	
Disease stage				
IIIA	35 (45.5)	24 (49.0)	11 (39.3)	0.285
IIIB	34 (44.2)	22 (44.9)	12 (42.9)	
IIIC	8 (10.4)	3 (6.1)	5 (17.9)	
Tumor histological type				
Squamous cell carcinoma	58 (75.3)	38 (77.6)	20 (71.4)	0.549
Non-squamous cell carcinoma	19 (24.7)	11 (22.4)	8 (28.6)	
Radiotherapy dose				
54-59 Gy	17 (22.1)	11 (22.4)	6 (21.4)	0.917
60-66 Gy	60 (77.9)	38 (77.6)	22 (78.6)	
Induction therapy cycles				
3-4 cycles	37 (48.1)	20 (40.8)	17 (60.7)	0.093
5-6 cycles	40 (51.9)	29 (59.2)	11 (39.3)	

†The chi-square test or Fisher’s exact test.

ECOG PS, Eastern Cooperative Oncology Group Performance Status.

### 3.2 Efficacy

At the cut-off period on Sep 15, 2022, the median follow-up was 22.7 months (range: 2.1–33.4). Fifteen out of 49 patients (30.6%) suffered from PD or death in cohort A, and 20 of 28 patients (71.4%) suffered from PD or death in cohort B. In cohort A, of the three patients (6.1%) who experienced distant metastasis, two and one experienced brain and bone metastases, respectively. In cohort B, one patient experienced brain metastasis, one had liver metastasis, and one had bone metastasis among the three patients (10.7%) with distant metastasis.

**Figure 1:**
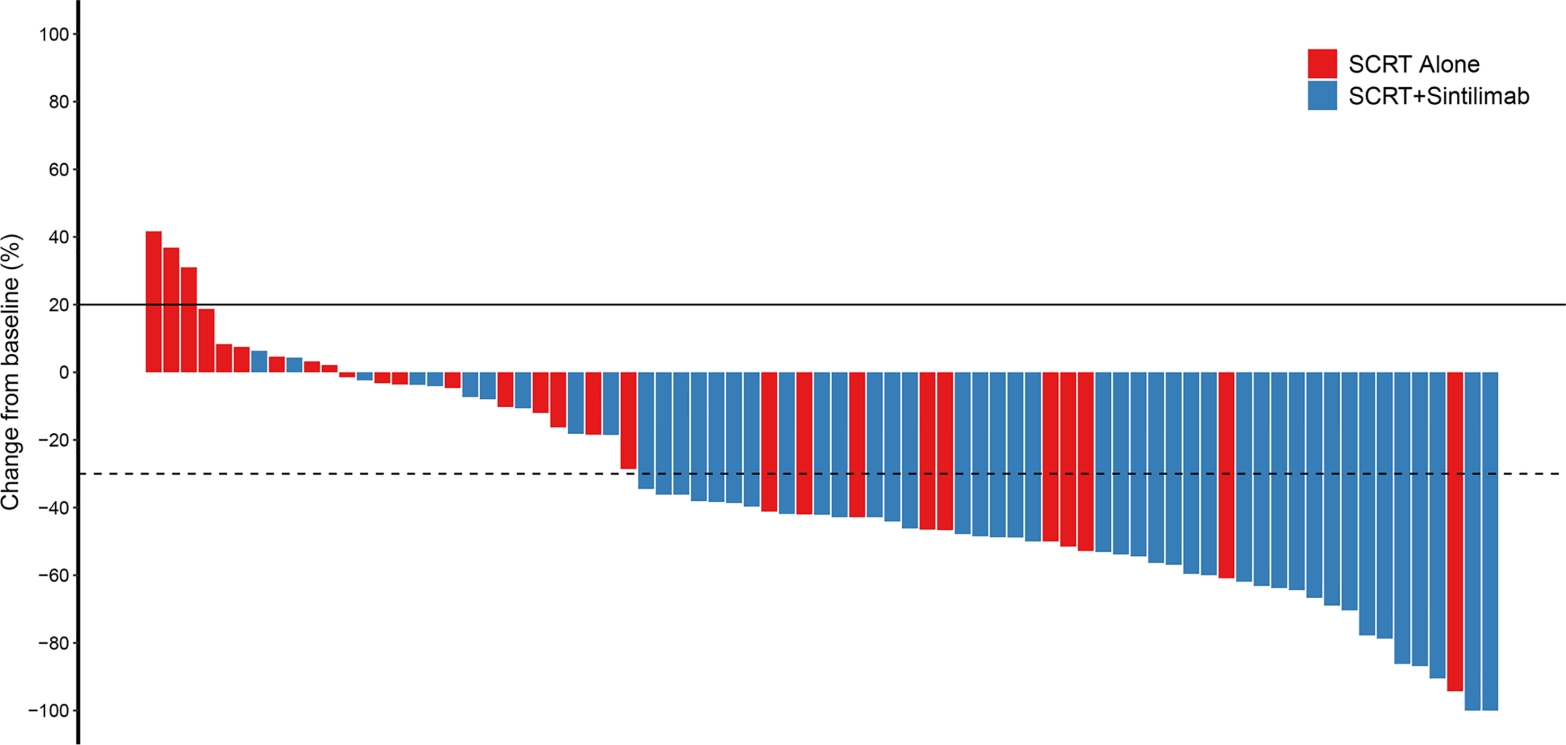
Change from baseline. Percent change in targeted lesion size from the baseline when the best overall response was observed for the tumor using RECIST. SCRT, sequential chemoradiotherapy; RECIST, response evaluation criteria in solid tumors.

The ORR was significantly higher in cohort A (79.6%, 95% CI 65.7–89.8) than in cohort B (35.7%, 95% CI 18.6–55.9) (p<0.001) (**Table 2**). Median DoR was not reached in cohort A (95%CI 20.2-NR) and was 17.8 months in cohort B (95%CI 12.1-NR). Among the patients whose best response was CR or PR, an ongoing response was observed in 82.8% and 72.0% of patients in cohort A and 80.0% and 48.0% of patients in cohort B at 12 and 18 months, respectively. **Figure 1** demonstrated the percent change in targeted lesion size from the baseline while observing the best overall response for the tumor using RECIST 1.1.

**Table 2:** Anti-tumor activity in all patients receiving treatment.

Response	Cohort A (N=49)	Cohort B (N=28)
Objective response		
Patients with response	39	10
% of patients (95% CI)	79.6 (65.7-89.8)	35.7 (18.6-55.9)
Best overall response, No. (%)		
Complete response	2 (4.1)	0 (0)
Partial response	37 (75.5)	10 (35.7)
Stable disease	10 (20.4)	15 (53.6)
Progressive disease	0 (0)	3 (10.7)
Duration of response, median (95% CI), mo†	NR (20.2-NR)	17.8 (12.1-NR)
Patients with response ≥12 mo, %	82.8	72.0
Patients with response ≥18 mo, %	80.0	48.0

†Analysed among patients who achieved an objective response.

**Figure 2:**
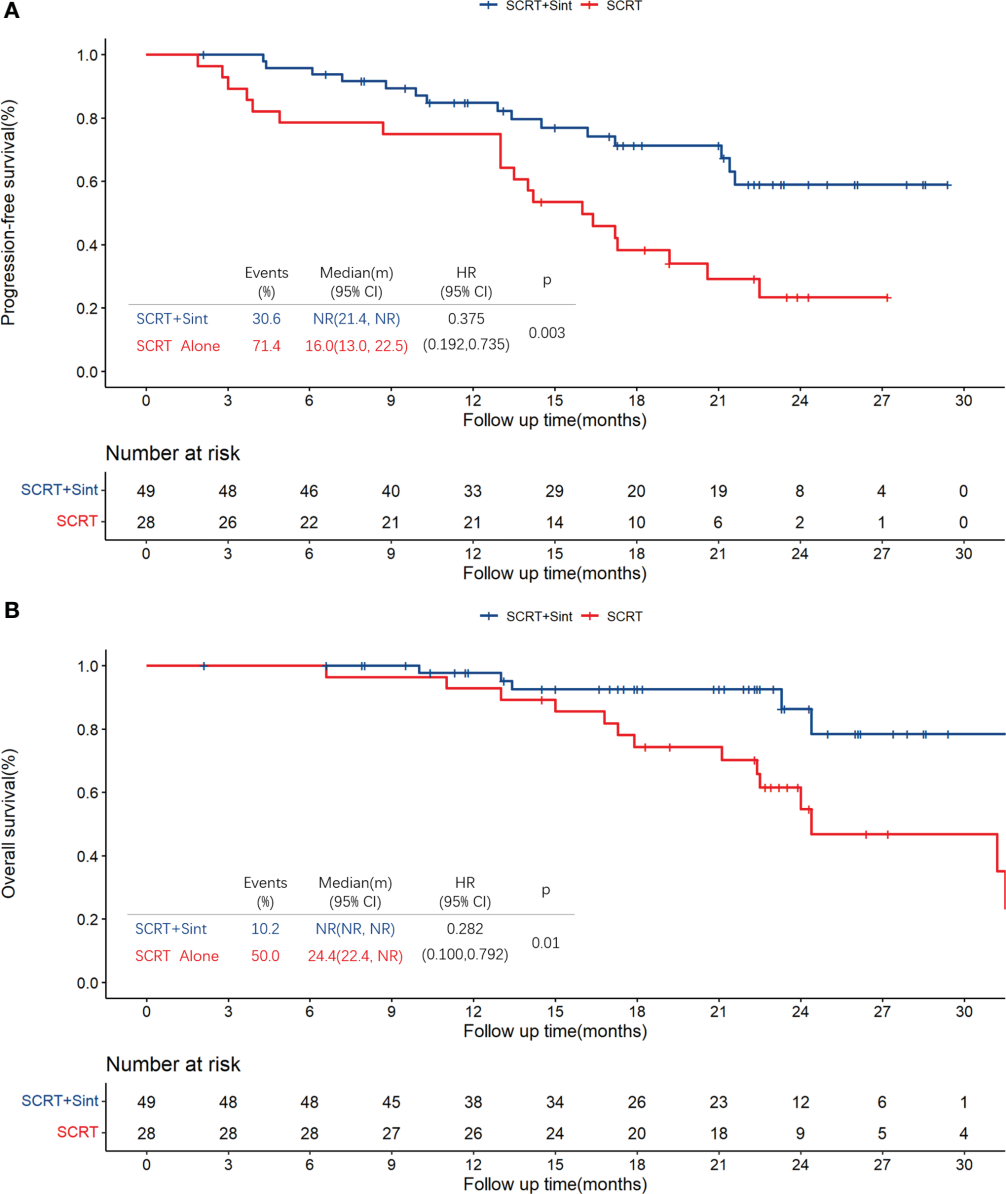
Kaplan-Meier plots for PFS **(A)** and OS **(B)** in all patients. PFS, progression-free survival; OS, overall survival; SCRT, sequential chemoradiotherapy; Sint, sintilimab.

**Figure 3:**
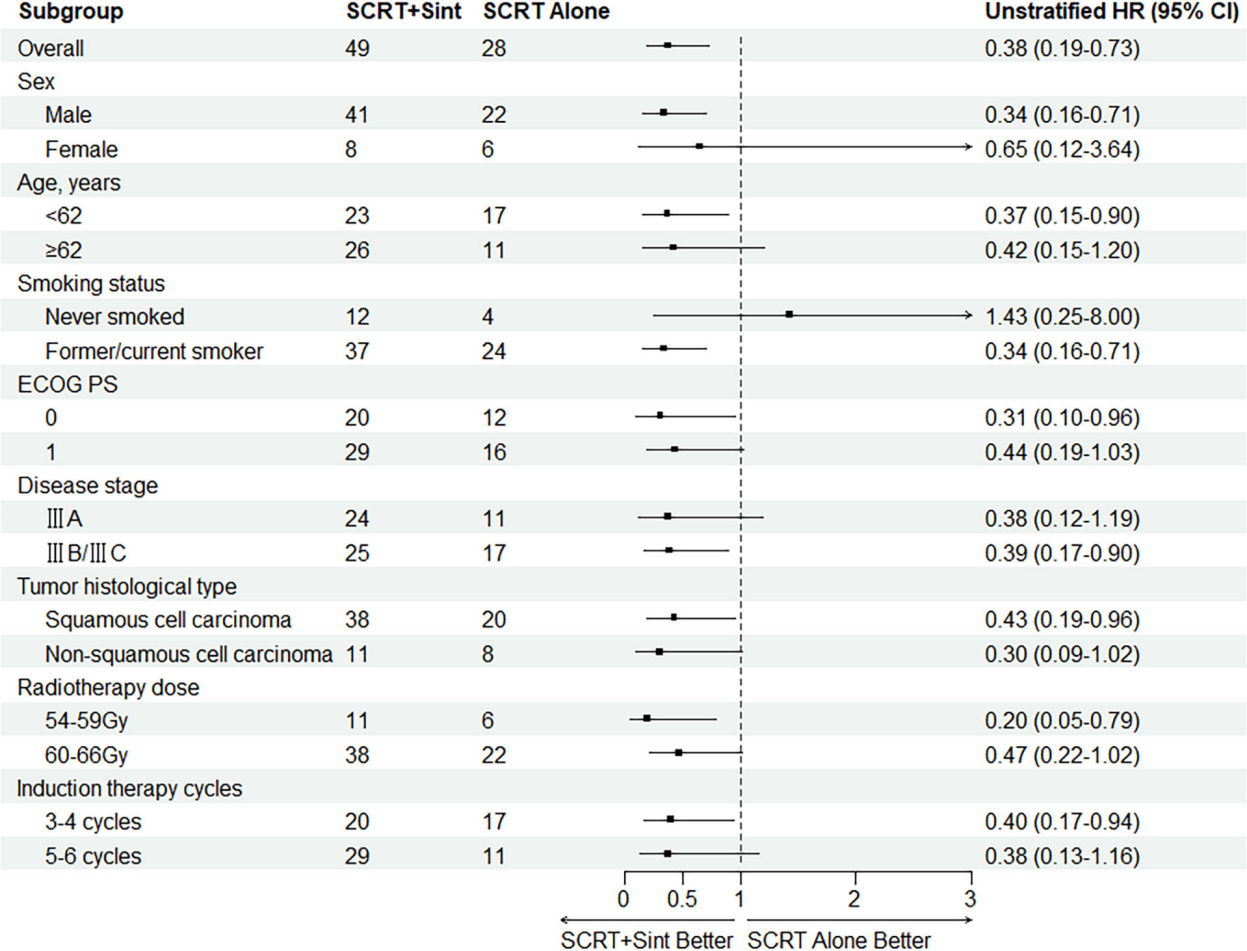
Subgroup analysis of PFS. SCRT, sequential chemoradiotherapy; Sint, sintilimab; ECOG PS, Eastern Cooperative Oncology Group Performance Status.

**Figure 4:**
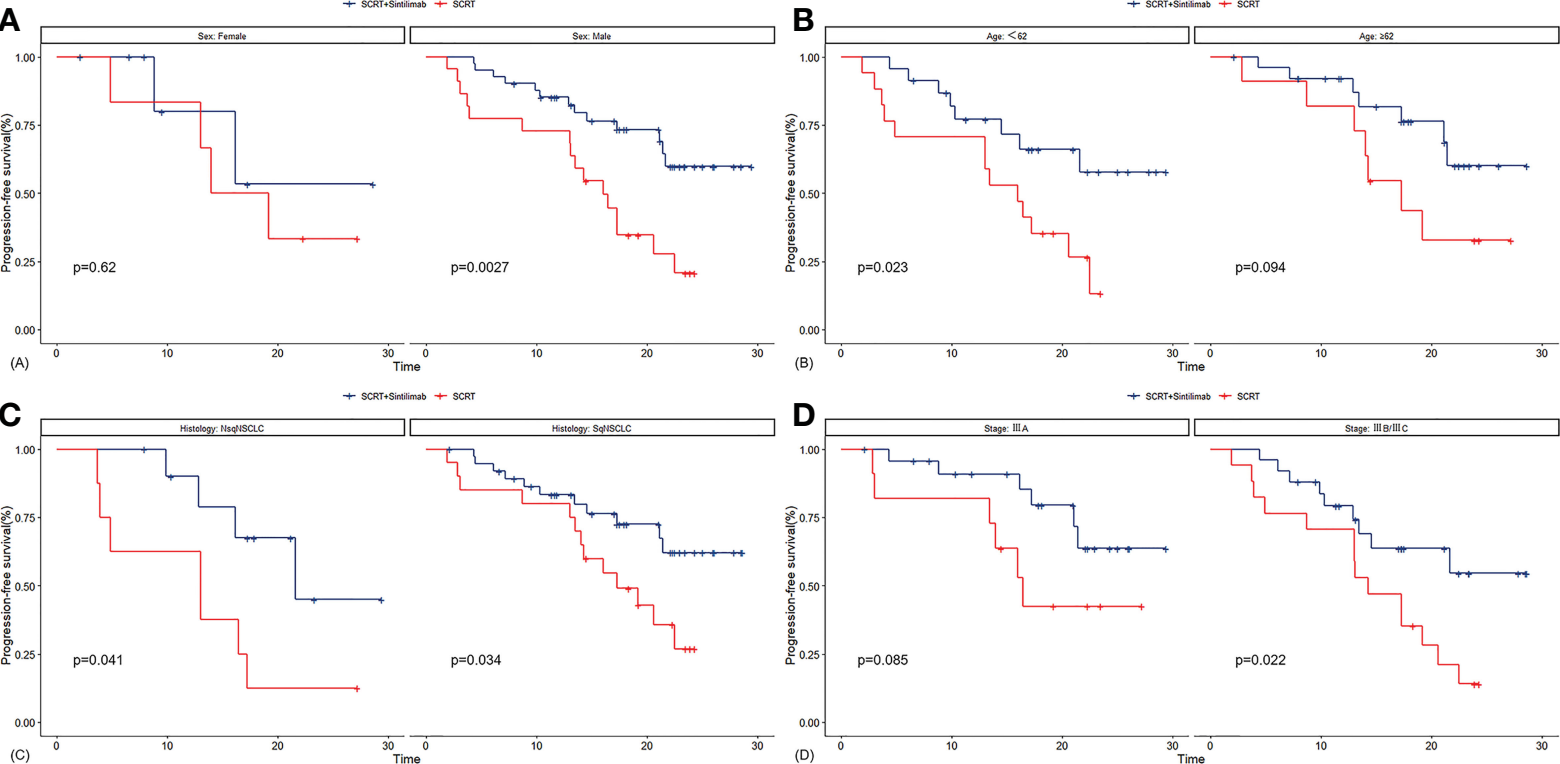
Kaplan-Meier plots for PFS in subgroups of sex **(A)**, age **(B)**, histology **(C)** and tumor stage **(D)**. SCRT, sequential chemoradiotherapy; NsqNSCLC, sqNSCLC, non-squamous non-small-cell lung cancer; sqNSCLC, squamous non-small-cell lung cancer.

Median PFS was significantly longer in cohort A than in cohort B (NR [95% CI 21.4–NR] vs. 16.0 months [13.0–22.5]; HR 0.375, 95% CI 0.192–0.735; p=0.003; **Figure 2A**). The PFS rates at 12 and 18 months were 84.8% (95% CI 75.0–95.9) and 71.3% (95% CI 58.7–86.7) in cohort A and 75.0% (95% CI 60.6–92.9) and 38.3% (95% CI 23.7–61.7) in cohort B, respectively. The sintilimab-combination cohort exhibited a superior PFS benefit than the SCRT-alone cohort across most prespecified subgroups (**Figure 3**). Median OS was not reached in cohort A (95% CI NR-NR) and was 24.4 months in cohort B (95% CI 22.4-NR). The OS was significantly longer in cohort A than in cohort B (HR 0.282; 95% CI 0.100-0.792; p=0.01; **Figure 2B**). The OS rates at 12 and 18 months were 97.7% (95% CI 93.4–100.0) and 92.5% (95% CI 84.7–100.0) in cohort A, and 92.9% (95% CI 83.8–100.0) and 74.4% (95% CI 59.7–92.7) in cohort B, respectively. **Figure 4**, **Supplementary Figure 2** depicted the Kaplan-Meier plots for the PFS comparison of treatment differences in each subgroup.

### 3.3 Safety

AEs of any grade were reported in 45 patients (91.8%) in cohort A and 24 patients (85.7%) in cohort B. AEs of grade 3 or 4 were reported in 19 patients (38.8%) and seven patients (25.0%) in two cohorts. AEs leading to any treatment discontinuation were reported in seven patients (14.3%) in cohort A and two patients (7.1%) in cohort B. AEs leading to death were reported in one patient (2.0%) and zero in cohorts A and B, respectively. Anemia was the most common AE of any grade in both cohorts (59.2% and 53.6%, respectively). The most common AEs of grade 3 or 4 were pneumonitis or immune-mediated pneumonitis (10.2%) and decreased neutrophil count (10.2%) in cohort A, and decreased white blood cell count (10.7%) in cohort B.

Grade 3 or 4 pneumonitis or immune-mediated pneumonitis, radiation pneumonitis, and pneumonia occurred in five (10.2%), four (8.2%), and two (4.1%) patients in cohort A, and zero, two (7.1%), and two (7.1%) in cohort B, respectively. The most common AEs leading to any treatment discontinuation were pneumonitis or immune-mediated pneumonitis in cohort A, with four patients (8.2%) discontinuing treatment due to pneumonitis or immune-mediated pneumonitis, including one patient, who exhibited PD after discontinuation. AEs leading to any treatment discontinuation occurred in only two patients (7.1%), one with pneumonia and another with a decreased neutrophil count in cohort B. Among all the patients, the only AE leading to death was immune-mediated pneumonitis in cohort A. Pneumonitis or immune-mediated pneumonitis, radiation pneumonitis and pneumonia of any grade occurred in nine (18.4%), nine (18.4%), and eight (16.3%) patients in cohort A, and two (7.1%), five (17.9%) and four (14.3%) in cohort B, respectively (**Table 3**).

**Table 3:** Adverse events of any cause.

Event	Cohort A		Cohort B	
	Any Grade	Grade 3 or 4	Any Grade	Grade 3 or 4
Any event	45 (91.8)	19 (38.8)	24 (85.7)	7 (25.0)
AE leading to any treatment discontinuation	7 (14.3)	7 (14.3)	2 (7.1)	2 (7.1)
AE leading to death	1 (2.0)	1 (2.0)	0	0
Anemia	29 (59.2)	1 (2.0)	15 (53.6)	1 (3.6)
White blood cell count decreased	22 (44.9)	4 (8.2)	13 (46.4)	3 (10.7)
Neutrophil count decreased	20 (40.8)	5 (10.2)	13 (46.4)	1 (3.6)
Fatigue	18 (36.7)	0	9 (32.1)	0
Decreased appetite	17 (34.7)	0	10 (35.7)	0
Nausea	17 (34.7)	0	11 (39.3)	0
Pruritus	14 (28.6)	0	0	0
Rash	13 (26.5)	1 (2.0)	1 (3.6)	0
Weight decreased	12 (24.5)	0	2 (7.1)	0
Pneumonitis or immune-mediated pneumonitis†	9 (18.4)	5 (10.2)	2 (7.1)	0
Radiation pneumonitis	9 (18.4)	4 (8.2)	5 (17.9)	2 (7.1)
Hypothyroidism	9 (18.4)	1 (2.0)	0	0
Constipation	9 (18.4)	0	4 (14.3)	0
Pneumonia	8 (16.3)	2 (4.1)	4 (14.3)	2 (7.1)
Vomiting	8 (16.3)	0	6 (21.4)	0
Blood creatinine increased	8 (16.3)	0	1 (3.6)	0
Platelet count decreased	7 (14.3)	2 (4.1)	4 (14.3)	2 (7.1)
Aspartate aminotransferase increased	7 (14.3)	1 (2.0)	3 (10.7)	0
Diarrhea	7 (14.3)	0	3 (10.7)	0
Alanine aminotransferase increased	6 (12.2)	2 (4.1)	2 (7.1)	0
Pyrexia	5 (10.2)	0	0	0

AE, adverse events.†The distinction of pneumonitis or immune-mediated pneumonitis, radiation pneumonitis and pneumonia was further clarified in **Supplementary File 1.**

## 4 Discussion

The results of this retrospective study demonstrated that concurrent sintilimab with SCRT had a significantly higher ORR and significant PFS benefit compared to that with SCRT alone as the first-line therapy in unresectable stage III NSCLC patients. The sintilimab-combination regimen possessed manageable safety profiles.

In this study with a median follow-up time of 22.7 months, median PFS and median OS were not reached in the sintilimab-SCRT combination cohort (cohort A). They were significantly longer than those in the SCRT-alone cohort (cohort B). Notably, a PFS benefit trend favoring the sintilimab-combination regimen was observed in most subgroups, such as stage IIIA/IIIB, stage IIIC and squamous/non-squamous cell carcinoma. The PFS rates in cohort A at 12 and 18 months were 84.8% and 71.3%, respectively. The two cohorts in the KEYNOTE-799 study using concurrent pembrolizumab with CCRT in unresectable locally advanced NSCLC had PFS rates of 67.1% and 71.6% at 12 months (23). In the ETOP NICOLAS study, unresectable locally advanced NSCLC patients were treated with concurrent nivolumab with CCRT. Moreover, a median PFS of 12.7 months, a median OS of 38.8 months, and a PFS rate of 53.7% at 12 months were reported in the ETOP NICOLAS study (35). In the phase I clinical study of 21 patients with unresectable locally advanced NSCLC treated with concurrent pembrolizumab with CCRT, a median PFS of 18.7 months and PFS rates of 81.0% and 69.7% were achieved at 12 and 18 months, respectively (36). Moreover, in our study, the ORR in the sintilimab-SCRT combination cohort reached 79.6%, 43.9% higher than in the SCRT alone cohort. Among the patients exhibiting an objective response in cohort A, 82.8% and 72.0% had an ongoing response at 12 and 18 months, and the median DoR was not reached. This demonstrates the satisfactory ability of the sintilimab-SCRT combination therapy for tumor disease control. Survival results from this study were similar to the results of currently published trials researching the concurrent PD1/PDL1 inhibitor combined with CCRT, including the KEYNOTE-799, ETOP NICOLAS study, and the phase I study by Jabbour (KEYNOTE-799: ORR: 70.5%/70.6%, DOR: NR; ETOP NICOLAS: ORR: 73.4%, DOR: 11 months; phase I studies of Jabbour: ORR: 89%) (23, 35, 36).

SCRT was used as the control group due to more clinical practice representativeness in China. Before the advent of the PD1/PDL1 inhibitors for treating of locally advanced unresectable NSCLC, sufficient trial data established significantly better survival in patients receiving CCRT than those receiving SCRT (6, 30). Most scholars believed that using chemotherapy and radiotherapy in the CCRT strategy was potentially advantageous due to early synergistic effects in the local control of tumors. This effectively reduces the probability of local progression, enabling patients to obtain better survival benefits compared to SCRT. However, with the use of concurrent PD1/PDL1 inhibitor for the treatment of unresectable locally advanced NSCLC, the efficacy of SCRT can be significantly improved. In this study, concurrent sintilimab with chemotherapy resulted in strong local control over tumors during induction therapy. Several studies showed that chemotherapy could result in immunological effects by enhancing the cross-presentation of tumor antigens, reducing T-regulatory cell activity, and inducing the expression of PD-L1 on tumor cells, thereby synergistically enhancing the anti-tumor activity of the PD1/PDL1 inhibitor (37–40). In ORIENT-11 and ORIENT-12 studies, patients with advanced or metastatic non-squamous and squamous NSCLC who received chemotherapy plus sintilimab exhibited a significant survival benefit than those who received chemotherapy plus placebo (32, 33). The strong synergy between PD1/PDL1 inhibitors and chemotherapeutic drugs will likely be the basis for treating future patients with unresectable stage III NSCLC with concurrent PD1/PDL1 inhibitors with SCRT. The survival data results obtained in this study provided reference and the possibility for large-scale clinical trial design.

In this study, the use of the sintilimab-SCRT combination demonstrated tolerable toxicity and a manageable safety profile. The incidence of grade 3 or 4 AEs in cohort A was 13.8% higher than in cohort B, but most AEs were manageable. Decreased neutrophil count and decreased white blood count were the most common hematological grade 3 or 4 AEs in cohorts A and B, respectively. However, they rarely led to treatment discontinuation or death due to the timely administration of drugs, including granulocyte colony-stimulating factors for treatment or prevention.

The incidence and severity of pneumonitis are currently the focus of the debate on combining PD1/PDL1 inhibitors and chemoradiotherapy. Concurrent PD1/PDL1 inhibitor combined with chemoradiotherapy increase pulmonary toxicity, leading to treatment discontinuation and impacting patient survival (23, 41). The incidence of pneumonitis or immune-mediated pneumonitis, radiation pneumonitis, and pneumonia of any grade was not more than 20%. Additionally, the incidence of radiation pneumonitis and pneumonia of grade 3 or 4 was not more than 10% in the two cohorts. However, immune-mediated pneumonitis was the most common AE of grade 3 or 4 in cohort A, followed by radiation pneumonitis. Immune-mediated pneumonitis resulted in four treatment discontinuation cases and one death from respiratory failure. In contrast, the only case of AE resulting in treatment discontinuation was pneumonia in cohort B, and no deaths from AEs were observed. The incidence of grade 3 or 4 pneumonitis or immune-mediated pneumonitis in cohort A was 10.2% higher than in cohort B. In contrast, grade 3 or 4 radiation pneumonitis and pneumonia incidence were similar in the two cohorts. The incidence of any grade pneumonitis or immune-mediated pneumonitis in cohort A was 11.3% higher than in cohort B. Moreover, the incidence of any grade radiation pneumonitis and pneumonia was similar in the two cohorts. In the GEMSTONE-301 study, grade 3 or 4 immune-mediated pneumonitis was reported in less than 4% of the population, and no grade 3 or 4 radiation pneumonitis could be observed (20). Therefore, concurrent PD1/PDL1 inhibitors with SCRT could increase the incidence of any grade or severe immune-mediated pneumonitis compared to that observed with SCRT alone, but the safety profile is manageable.

Pneumonitis incidence in our study was consistent with the KEYNOTE-799 and the ETOP NICOLAS studies and significantly higher than in the PACIFIC trial and the GEMSTONE-301 study (18, 20, 23, 28). This indicated that concurrent immunotherapy is riskier than consolidation immunotherapy regardless of the chemoradiotherapy sequence. The KEYNOTE-799 study reported estimated incidence of 22% and 6% for immune-mediated pneumonitis of any grade and grade 3 or 4, respectively, as well as 17.9%/7.8% (cohort A/B in KEYNOTE-799) and 1.8%/1.0% for radiation pneumonitis of any grade and grade 3 or 4, respectively (23). In the ETOP NICOLAS study, the incidence of pneumonitis of any grade and grade 3 or 4 were 42.5% and 11.7%, respectively (28). Notably, we did not observe the expected reduction in the incidence of pneumonitis in concurrent sintilimab with SCRT therapy in this study, compared to that observed with concurrent PD1 inhibitor with CCRT in the KEYNOTE-799 study. This may be because the patients included in our study were from the real world, whose basic cardiopulmonary status was worse than those in other prospective clinical studies, becoming prone to pneumonitis.

Despite reliable data and rigorous research, this retrospective study has several limitations. First, the allocation of patients in cohort A and cohort B was not based on the principle of randomization, and whether patients received the sintilimab-SCRT combination regimen was largely affected by their subjective will and economic ability, which inevitably resulted in a bias. In addition, the small sample size was a limitation in this study, as it could cause inaccuracies during the survival analysis of individual subgroups. Finally, the absence of information regarding the gene mutation status and PDL1 expression in the real world prevented us from exploring these critical aspects in our study. Further studies must be conducted on a large scale to consider these aspects. Despite these limitations, this study is the first to investigate the efficacy and safety of the combination of sintilimab and SCRT in patients with unresectable stage III NSCLC in China and provides a reference for clinicians aiming to explore a novel regimen for these patients.

In conclusion, this study demonstrated that concurrent sintilimab with SCRT as a first-line therapy could significantly improve ORR and prolong PFS in unresectable stage III NSCLC patients from China. No new unexpected safety signals were observed. The results of this study could help establish a novel regimen for this patient population.

## Data availability statement

The original contributions presented in the study are included in the article/**Supplementary Material**. Further inquiries can be directed to the corresponding author.

## Ethics statement

The studies involving human participants were reviewed and approved by the First Hospital of Jilin University Ethics Committee. The patients/participants provided their written informed consent to participate in this study. Written informed consent was obtained from the individual(s) for the publication of any potentially identifiable images or data included in this article.

## Author contributions

ST analyzed the data and wrote the manuscript. XC reviewed and edited the manuscript. DZ, JG, and HZ enrolled the patients and collected the data. CC and ZGL supervised the study. YZ analyzed and assembled the data. ZLL designed the study and revised the manuscript. All authors contributed to the article and approved the submitted version.

## Supplementary material

Supplementary Figure 1 Flow chart for patient enrolment. NSCLC, non-small-cell lung cancer; SCRT, sequential chemoradiotherapy.

Supplementary Figure 2 Kaplan-Meier plots for PFS in subgroups of smoking status **(A)**, ECOG performance status **(B)**, radiotherapy dose **(C)** and induction therapy cycles **(D)** SCRT, sequential chemoradiotherapy; ECOG, Eastern Cooperative Oncology Group.
