# Supplementary figures and images for: Concurrent sintilimab with sequential chemoradiotherapy for unresectable, stage III non-small cell lung cancer: a retrospective study
Source: Front Oncol. 2023 Apr 20;13:1129989. doi: 10.3389/fonc.2023.1129989 (PMC10157220; doi:10.3389/fonc.2023.1129989)

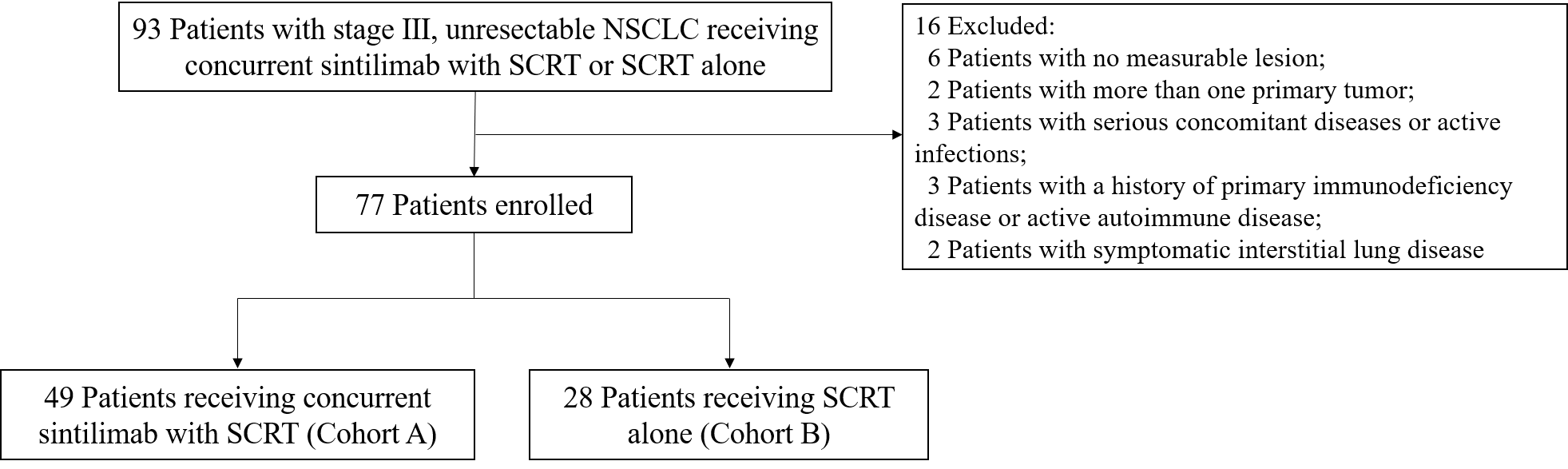

Supplement: Supplementary Figure 1 — Flow chart for patient enrolment. NSCLC, non-small-cell lung cancer; SCRT, sequential chemoradiotherapy. [file Image_1.tif]

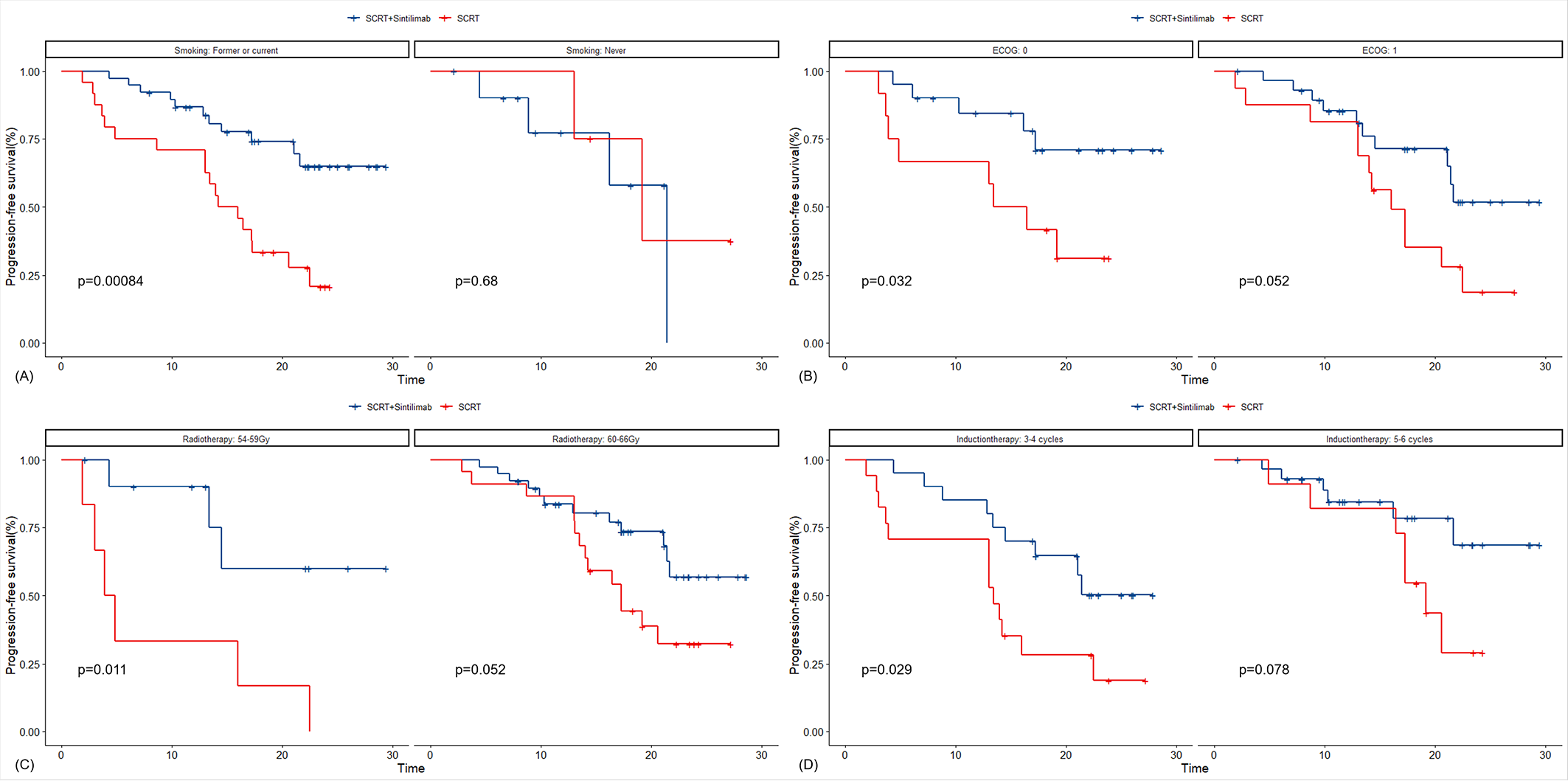

Supplement: Supplementary Figure 2 — Kaplan-Meier plots for PFS in subgroups of smoking status (A), ECOG performance status (B), radiotherapy dose (C) and induction therapy cycles (D) SCRT, sequential chemoradiotherapy; ECOG, Eastern Cooperative Oncology Group. [file Image_2.tif]
