# Supplementary material for: Concurrent sintilimab with sequential chemoradiotherapy for unresectable, stage III non-small cell lung cancer: a retrospective study
Source: Front Oncol. 2023 Apr 20;13:1129989. doi: 10.3389/fonc.2023.1129989 (PMC10157220; doi:10.3389/fonc.2023.1129989)
Supplement: Supplementary file 3 [file Presentation_1.pdf]

We distinguish the nature of pneumonitis based on the following identification points in this study:

Pneumonia (infectious pneumonitis): fever, rigor, cough, increase in white blood count, C-reactive protein and procalcitonin, sputum culture positive, blood culture positive, radiologic changes, response to antibiotics;

Radiation pneumonitis (radiotherapy-induced pneumonitis): cough, dyspnea, timing and history of radiation therapy, radiologic features (ground glass opacities, airspace consolidation or linear streaks within the irradiated field), response to steroids and antibiotics;

Pneumonitis or immune-mediated pneumonitis: cough, dyspnea, radiologic features (ground-glass opacities or patchy areas of consolidation which are often seen in the periphery; reticular markings, traction bronchiectasis, and ground-glass opacities are seen mostly in lower zones), response to steroids and antibiotics.
